# Transcriptomic Signatures of End-Stage Human Dilated Cardiomyopathy Hearts with and without Left Ventricular Assist Device Support

**DOI:** 10.3390/ijms23042050

**Published:** 2022-02-12

**Authors:** Mihir Parikh, Saumya Shah, Ratnadeep Basu, Konrad S. Famulski, Daniel Kim, John C. Mullen, Philip F. Halloran, Gavin Y. Oudit

**Affiliations:** 1 Division of Cardiology, Department of Medicine, Faculty of Medicine and Dentistry, University of Alberta, Edmonton, AB T6G 2R3, Canada; parikhmihirp@outlook.com (M.P.); saumya@ualberta.ca (S.S.); ratnadee@ualberta.ca (R.B.); dk@ualberta.ca (D.K.); 2 Mazankowski Alberta Heart Institute, Edmonton, AB T6G 2R3, Canada; jmullen@ualberta.ca; 3 Division of Nephrology, Department of Medicine, Faculty of Medicine and Dentistry, University of Alberta, Edmonton, AB T6G 2R3, Canada; konrad.famulski@ualberta.ca (K.S.F.); phil.halloran@ualberta.ca (P.F.H.); 4 Division of Cardiac Surgery, Department of Medicine, Faculty of Medicine and Dentistry, University of Alberta, Edmonton, AB T6G 2R3, Canada; 5 Department of Physiology, Faculty of Medicine and Dentistry, University of Alberta, Edmonton, AB T6G 2R3, Canada

**Keywords:** translational studies, gene expression and regulation, cardiomyopathy, heart failure, reverse remodelling, left ventricular assist device

## Abstract

Left ventricular assist device (LVAD) use in patients with dilated cardiomyopathy (DCM) can lead to a differential response in the LV and right ventricle (RV), and RV failure remains the most common complication post-LVAD insertion. We assessed transcriptomic signatures in end-stage DCM, and evaluated changes in gene expression (mRNA) and regulation (microRNA/miRNA) following LVAD. LV and RV free-wall tissues were collected from end-stage DCM hearts with (*n* = 8) and without LVAD (*n* = 8). Non-failing control tissues were collected from donated hearts (*n* = 6). Gene expression (for mRNAs/miRNAs) was determined using microarrays. Our results demonstrate that immune response, oxygen homeostasis, and cellular physiological processes were the most enriched pathways among differentially expressed genes in both ventricles of end-stage DCM hearts. LV genes involved in circadian rhythm, muscle contraction, cellular hypertrophy, and extracellular matrix (ECM) remodelling were differentially expressed. In the RV, genes related to the apelin signalling pathway were affected. Following LVAD use, immune response genes improved in both ventricles; oxygen homeostasis and ECM remodelling genes improved in the LV and, four miRNAs normalized. We conclude that LVAD reduced the expression and induced additional transcriptomic changes of various mRNAs and miRNAs as an integral component of the reverse ventricular remodelling in a chamber-specific manner.

## 1. Introduction

Dilated cardiomyopathy (DCM) is a common manifestation of end-stage heart disease, characterized by left ventricle (LV) dilation, systolic dysfunction, and heart failure (HF) [1,2]. DCM is currently the leading cause of cardiac transplantation in adults. The improved survival of patients with HF, coupled with the overall rise in the prevalence of heart diseases, has led to an increase in the number of patients with advanced HF [3,4]. This creates a supply–demand imbalance for cardiac transplantation, with the number of recipients far exceeding the number of available donor hearts. Left ventricular assist devices (LVAD) represents a critical therapy as bridge to transplant or recovery, and potentially as destination therapy in patients with contraindications for transplantation [5,6,7]. The REMATCH [8] and the INTrEPID [9] trial revealed superior survival rates in patients with LVAD over conventional therapy and provided convincing evidence for the use of LVAD as bridge to transplant or recovery and also as potential destination therapy [10]. Treatment with continuous-flow LVAD in patients with advanced HF improves the probability of survival, quality of life and functional capacity compared with a pulsatile device [11,12].

The hemodynamic alteration with LVAD triggers beneficial remodelling at multiple molecular and cellular levels, leading to improved systolic and diastolic function [6,7]. However, the LV and right ventricle (RV) respond differently to the unloading effects of LVAD. In fact, RV dysfunction is the predominant complication post-LVAD implantation and the major cause of morbidity and mortality in these patients, indicating unique differences in RV and LV remodelling [13,14]. The molecular and cellular changes that occur in the heart as a result of LVAD therapy can provide important insight into the differential ventricular response while also clearly identifying the therapeutic benefits of LVAD. Our study aimed to understand chamber-specific transcriptomic changes in explanted human hearts with DCM post-LVAD implantation. LV and RV samples from explanted DCM hearts with and without LVAD implantation, as well as non-failing control hearts, were used for a microarray-based analysis of global mRNA and microRNA (miRNA) expression. In this study, several genes and pathways involved in the pathogenesis of DCM, as well as distinct remodelling and gene regulation patterns in the LV and RV of DCM hearts in response to LVAD use, were identified.

## 2. Results

### 2.1. Patient Clinical Characteristics

The clinical characteristics of DCM patients with LVAD (VAD group) and without LVAD implantation (no LVAD, NVAD group) are summarized in Table 1. Both groups had similar demographics, physical exam assessments, past medical histories, medical therapies, laboratory values, and echocardiographic parameters. For VAD group, the median duration of LVAD support was 156 days (IQR: 131–268 days). Limited information was available on donors of non-failing hearts (NFC group). The median age of these six donors was 45 (IQR: 40–51) years old, and all of them had normal LV ejection fraction.

### 2.2. Histological Characteristics of Explanted DCM Hearts with and without LVAD

**Figure 1:**
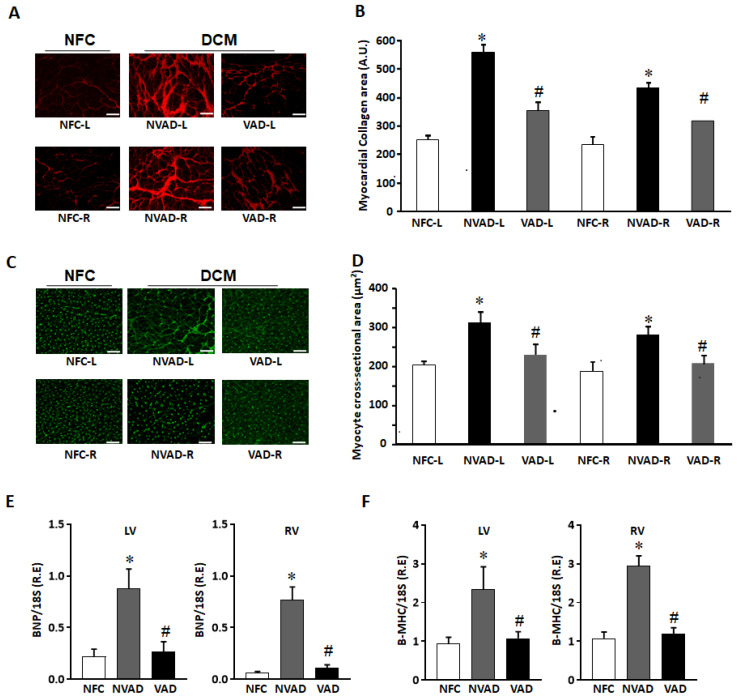
Histological characterization of non-failing control (NFC) and dilated cardiomyopathy (DCM) hearts. (**A**) Representative Picrosirius red staining images for collagen detection in NFC hearts, as well as DCM hearts with left ventricle assist device (VAD) and without LVAD support (NVAD). (**B**) Myocardial collagen content quantified from (**A**). (**C**) Representative Wheat germ agglutinin staining for NFC, DCM-VAD and DCM-NVAD hearts. (**D**) Cardiomyocyte cross-sectional area (CCA) quantified from (**C**). (**E**,**F**) Real-time quantitative PCR results showing the relative mRNA levels of hypertrophic markers brain natriuretic peptide (BNP) and β-myosin heavy chain (β-MHC) in NFC, NVAD, or VAD. *n* = 8, * *p* < 0.05 vs. NFC, # *p* < 0.05 vs. NVAD, unpaired two-tailed t-test. L, left; R, right. Scale bar represents 50 μm.

Increased cardiomyocyte hypertrophy and myocardial fibrosis are prominent features of adverse myocardial remodelling. Histological characteristics of VAD, NVAD and NFC hearts were analysed, with representative images shown in Figure 1. Fibrosis was increased in both the LV and RV of NVAD DCM hearts, at levels approximately 2-fold and 1.5-fold, respectively. Upon LVAD implantation, fibrosis was decreased in the VAD group compared to the NVAD group (Figure 1A,B). Similarly, cardiomyocyte cross-sectional area (CSA) was significantly larger in the LV and RV of NVAD DCM hearts (*p* < 0.05). Upon LVAD implantation, cardiomyocyte CSA was decreased in the VAD group compared to the NVAD group (Figure 1C,D). The cardiac enlargement was also confirmed in the NVAD by significantly up-regulated mRNA levels of the hypertrophic markers brain natriuretic peptide (BNP) and β-myosin heavy chain (β-MHC) compared to NFC. However, the levels of these biomarkers were reduced in VAD compared to NVAD (Figure 1E,F).

### 2.3. Pathological Genes of DCM Hearts

**Figure 2:**
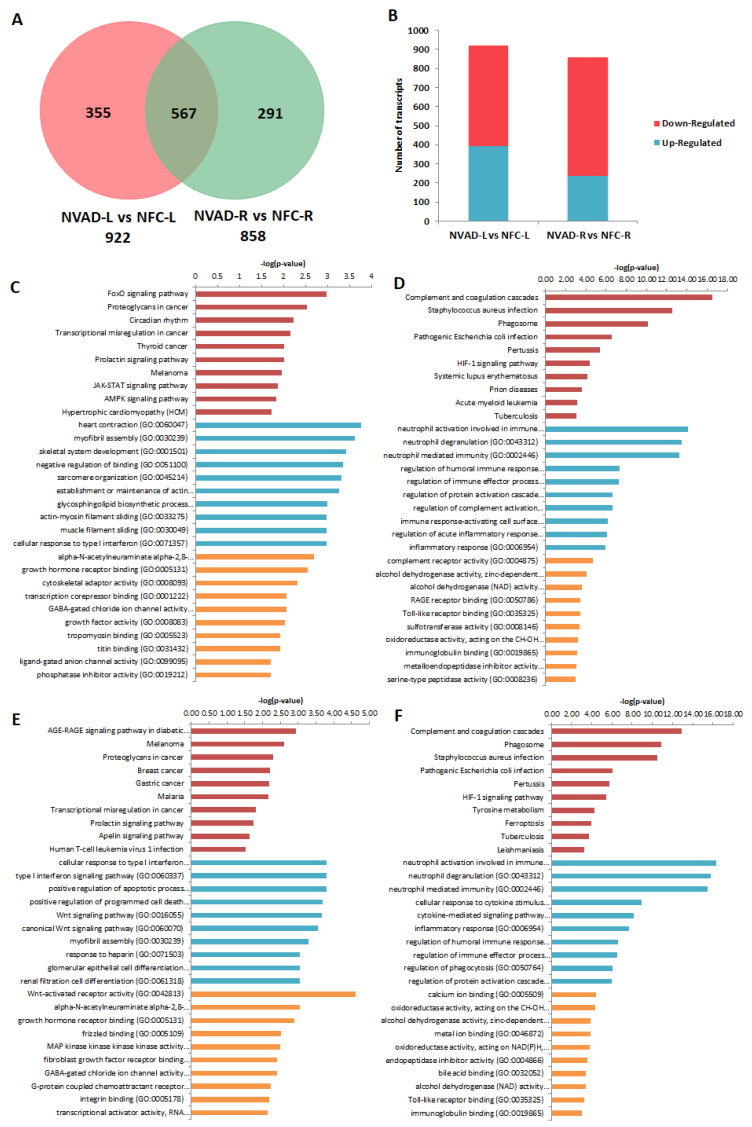
mRNA analysis of dilated cardiomyopathy (DCM) hearts without left ventricle assist device (NVAD) versus non-failing controls (NFC). (**A**) Number of significant differentially expressed transcripts in the left (L) and right (R) ventricles. (**B**) Number of significantly up- and down-regulated transcripts in the L and R ventricles. Gene ontology (GO) analysis for (**C**,**E**) up-regulated and (**D**,**F**) down-regulated differentially expressed genes in the (**C**,**D**) L and (**E**,**F**) R ventricles. The top ten Kyoto Encyclopedia of Genes and Genomes (KEGG) pathways (red), GO terms for biological processes (blue) and GO terms for molecular functions are shown (yellow).

The gene expression profile of DCM hearts without LVAD implantation (NVAD group) was compared to the gene expression profile of healthy hearts (NFC group) to identify transcripts and genes that were altered as a result of the disease. In the LV, 922 transcripts were differentially expressed (Figure 2A), 392 up-regulated and 530 down-regulated (Figure 2B), in the NVAD compared to the NFC groups. In the RV, 858 transcripts were differentially expressed (Figure 2A), 238 up-regulated and 620 down-regulated (Figure 2B), in the NVAD compared to the NFC groups. Of the differentially expressed transcripts found, 567 were commonly altered in both the LV and RV.

The most significantly enriched KEGG pathways and GO terms related to BP and MF for the up- and down-regulated genes encoding for the observed pathological transcripts are presented in Figure 2C–F. In the LV, 392 up-regulated pathological transcripts were the products of 238 genes (Figure 2C). The most enriched KEGG pathways for this gene set were involved in cellular physiology such as cell growth, proliferation, apoptosis, metabolism, and cell cycle regulation. The other enriched pathways included cardiomyocyte hypertrophy and circadian rhythm, while the majority of enriched BPs controlled myocardial structure and contractility. The enriched MFs reflect findings from pathways and BP analyses, with the majority of MFs involved in transcription, ion channels, cellular growth and muscle contraction. On the other hand, 530 down-regulated pathological transcripts were the products of 330 genes (Figure 2D). The majority of enriched KEGG pathways and BPs were involved in the immune response. Genes for the hypoxia-inducible factor 1 (HIF-1) pathway, which functions in oxygen homeostasis, were also enriched. With regard to MFs, the most enriched terms were related to innate immunity, regulation of the extracellular matrix (ECM), and cellular metabolism.

In the RV, 238 up-regulated pathological transcripts were the products of 157 genes (Figure 2E). Similar to up-regulated genes in the LV, the most significantly enriched KEGG pathways and BPs were involved in cellular physiological events. Genes involved in the apelin signalling pathway, which functions in key processes such as angiogenesis, cardiovascular function, cell proliferation, and energy regulation, were also enriched. MFs related to transcription, ion channels, cellular growth and metabolism were enriched. Genes involved in myocardial structure and contractility were not as enriched compared to what was seen in the LV. On the other hand, 620 down-regulated pathological transcripts were the products of 383 genes (Figure 2F). The enriched KEGG pathways, BPs and MFs of these genes were found to be similar to those for down-regulated genes in the LV (immune response, oxygen homeostasis). The lists of genes from all enrichment analyses, at least for the top 10 results for each category, are presented in Supplementary Material Table S1.

### 2.4. Gene Expression Changes in the LV and RV Post LVAD Implantation

**Figure 3:**
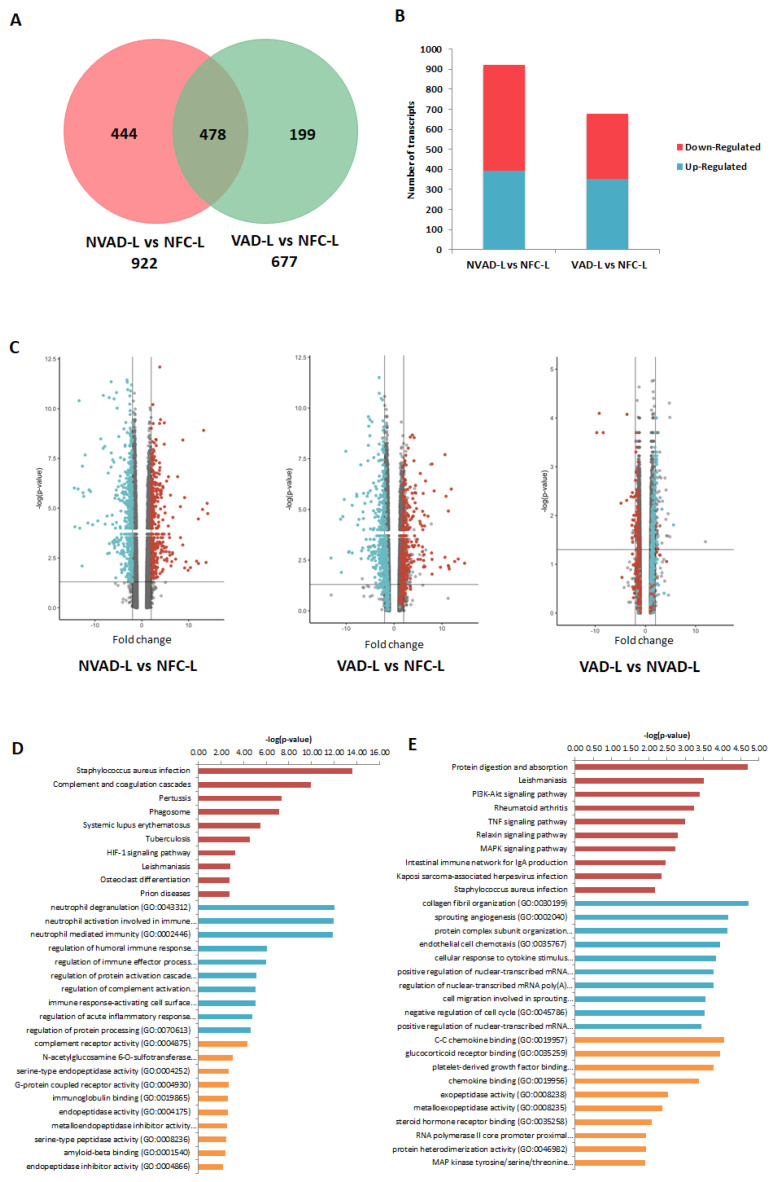
mRNA analysis of left ventricles (LV) from dilated cardiomyopathy (DCM) hearts with (VAD) and without left ventricle assist device (NVAD), using the non-failing controls (NFC) as reference. (**A**) Number of significant differentially expressed transcripts in the VAD and NVAD groups. (**B**) Number of significantly up- and down-regulated transcripts in the VAD and NVAD groups. (**C**) Volcano plots of microarray data from the VAD and NVAD groups. The LV DCM signature genes are marked (red: up-regulated, blue: down-regulated in NVAD group); grey vertical lines indicate two-fold fold-change values in either direction. Gene ontology (GO) analysis for (**D**) normalized genes in the LV following LVAD use and (**E**) additional differentially expressed LV genes following LVAD use. The top ten Kyoto Encyclopedia of Genes and Genomes (KEGG) pathways (red), GO terms for biological processes (blue) and GO terms for molecular functions are shown (yellow).

**Figure 4:**
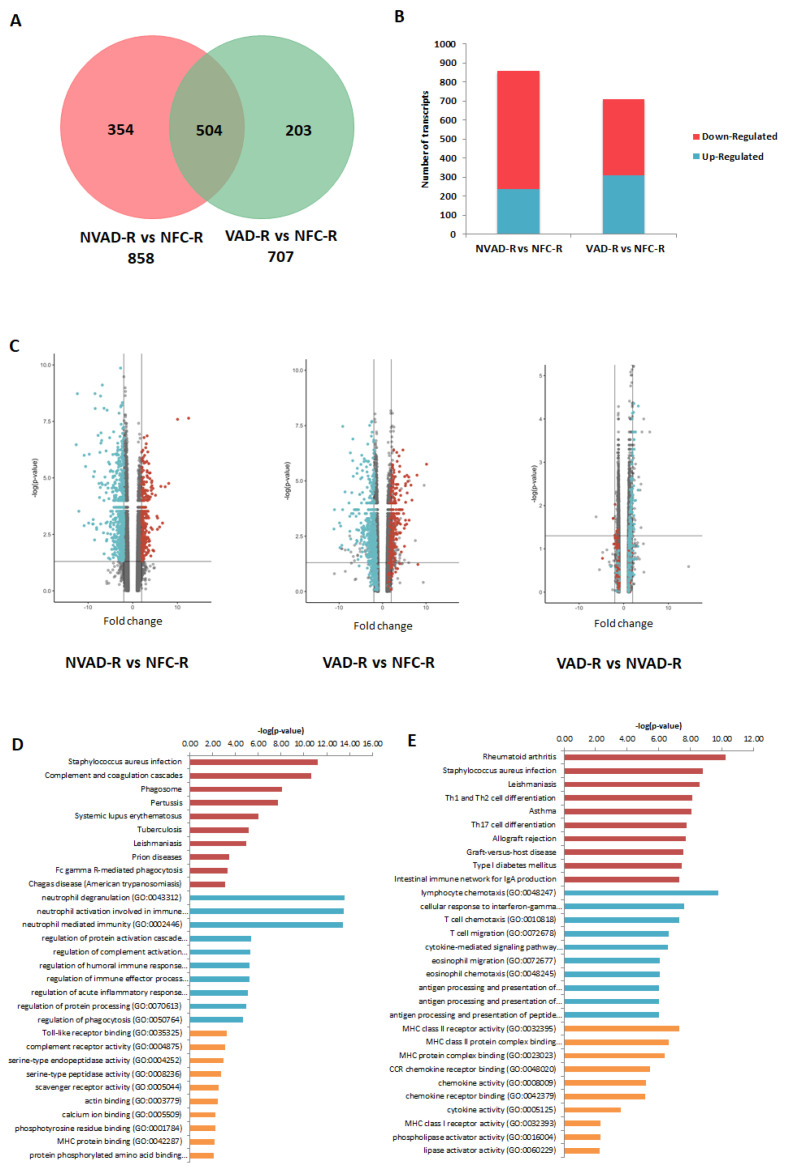
mRNA analysis of right ventricles (RV) from dilated cardiomyopathy (DCM) hearts with (VAD) and without left ventricle assist device (NVAD), using the non-failing controls (NFC) as reference. (**A**) Number of significant differentially expressed transcripts in the VAD and NVAD groups. (**B**) Number of significantly up- and down-regulated transcripts in the VAD and NVAD groups. (**C**) Volcano plots of microarray data from the VAD and NVAD groups. The RV DCM signature genes are marked (red: up-regulated, blue: down-regulated in NVAD group); grey vertical lines indicate two-fold fold-change values in either direction. Gene ontology (GO) analysis for (**D**) normalized genes in the RV following LVAD use and (**E**) additional differentially expressed RV genes following LVAD use. The top ten Kyoto Encyclopedia of Genes and Genomes (KEGG) pathways (red), GO terms for biological processes (blue) and GO terms for molecular functions are shown (yellow).

The LV and RV pathological transcripts found above were then tracked to see how their expressions changed upon LVAD implantation, as demonstrated by the VAD vs. NFC and VAD vs. NVAD comparisons (Figure 3). Results from the VAD vs. NFC comparisons revealed that of the 922 pathological transcripts identified in the LV, 617 (67%) showed a decrease in FC with LVAD use (compared to the FC in NVAD vs. NFC), and 130 (14%) showed no significant changes when compared to the NFC (Figure 3A–C). Of the 858 pathological transcripts identified in the RV, 493 (57%) showed a decrease in FC with LVAD use compared to without LVAD use, and 157 (18%) showed no changes compared to the NFC (Figure 4A–C). 

Comparisons between VAD and NVAD revealed significantly normalized transcripts (from pathological transcript lists) post-LVAD use in both the LV and RV. A transcript is considered normalized if the FC in the VAD vs. NVAD comparison shows the opposite direction of change (e.g., up-regulated) compared to the direction of change in the NVAD vs. NFC comparison (e.g., down-regulated). In the LV, 205 pathological transcripts (corresponding to 133 genes) were significantly normalized (Figure 3C). Enrichment analysis of these genes suggests that they function in immunity-related processes, oxygen homeostasis, as well as in the regulation of the ECM (Figure 3D). In the RV, 116 pathological transcripts (corresponding to 79 genes) were significantly normalized (Figure 4C). Enrichment analysis of these genes suggests that they also function in immunity (Figure 4D). Our results are consistent with increased apoptosis and cell death of cardiomyocytes and up-regulation of inflammatory cascade as known drivers of advanced HF.

Besides the normalization of pathological genes identified from our NVAD vs. NFC comparison, LVAD use also induced additional changes in gene expression as shown in the VAD vs. NVAD comparison. In the LV, 56 transcripts (corresponding to 36 genes) were significantly altered post-LVAD use. Enrichment analysis showed that the most enriched processes were involved in ECM remodelling, immune response, cellular physiological processes and angiogenesis (Figure 3E). In the RV, 23 transcripts (corresponding to 16 genes) were significantly altered post-LVAD use. Enrichment analysis showed that the majority of these genes were involved in immune system-related processes (Figure 4E). The gene lists for all the enrichment analyses in this section, at least for the top 10 results for each category, are presented in Supplementary Material Table S2.

### 2.5. Pathological miRNAs of DCM Hearts

**Figure 5:**
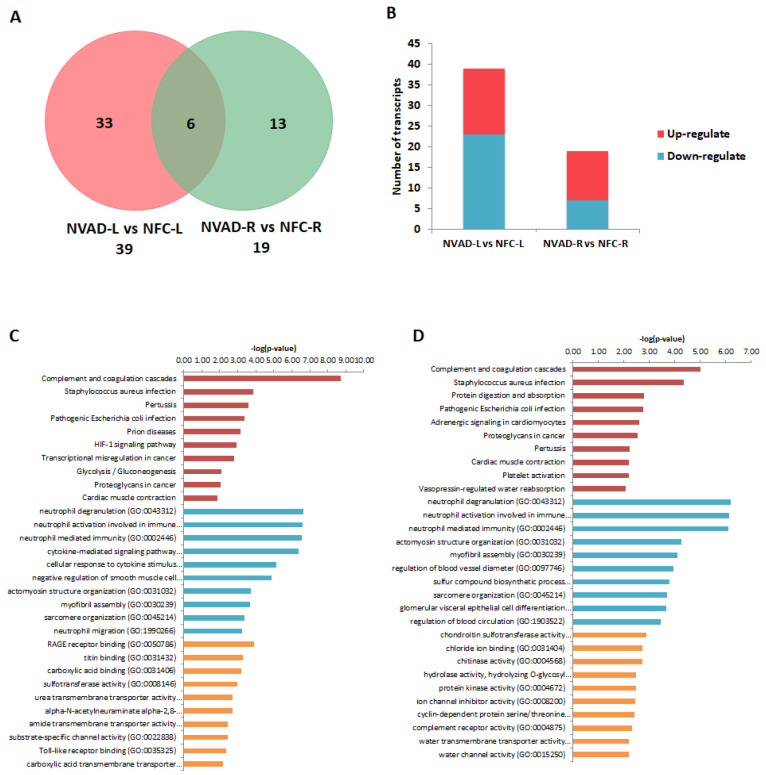
miRNA analysis of dilated cardiomyopathy (DCM) hearts without left ventricle assist device (NVAD) versus non-failing controls (NFC). (**A**) Number of significant differentially expressed miRNAs in the left (L) and right (R) ventricles. (**B**) Number of significantly up- and down-regulated miRNAs in the L and R ventricles. Gene ontology (GO) analysis for target genes of the concordant miRNAs in the (**C**) L and (**D**) R ventricles. The top ten Kyoto Encyclopedia of Genes and Genomes (KEGG) pathways (red), GO terms for biological processes (blue) and GO terms for molecular functions are shown (yellow).

The miRNA expression profile of the NVAD group was compared to that of the NFC group to identify miRNAs altered in DCM, which was termed pathological miRNAs. The target transcripts (and corresponding genes) of these pathological miRNAs were then compared to the pathological transcript/gene lists from our earlier mRNA analyses. This would determine whether the changes in mRNA expression we previously observed could be attributed to changes at the miRNA level. In the LV, 39 miRNAs were differentially expressed (Figure 5A), 16 up-regulated and 23 down-regulated (Figure 5B), in the NVAD compared to the NFC group. Of these 39 miRNAs, 18 regulate transcripts and genes that were altered in our earlier NVAD vs. NFC comparison (Table 2). Enrichment analysis of these genes showed that they are involved in immunity, oxygen homeostasis, cardiac muscle contraction, and cellular processes such as energy regulation, growth, proliferation, and apoptosis (Figure 5C). In the RV, 19 miRNAs were differentially expressed (Figure 5A), 12 up-regulated and seven down-regulated (Figure 5B), in the NVAD compared to the NFC group. Of these 19 miRNAs, 13 regulate transcripts and genes that were altered in our earlier NVAD vs. NFC comparison (Table 3). Enrichment analysis of these genes showed that they are involved in immunity, adrenergic signalling in cardiomyocytes, cardiac muscle contraction, and cellular processes such as energy regulation, growth, proliferation and apoptosis (Figure 5D).

### 2.6. miRNA Expression Changes in the LV and RV Post LVAD Implantation

**Figure 6:**
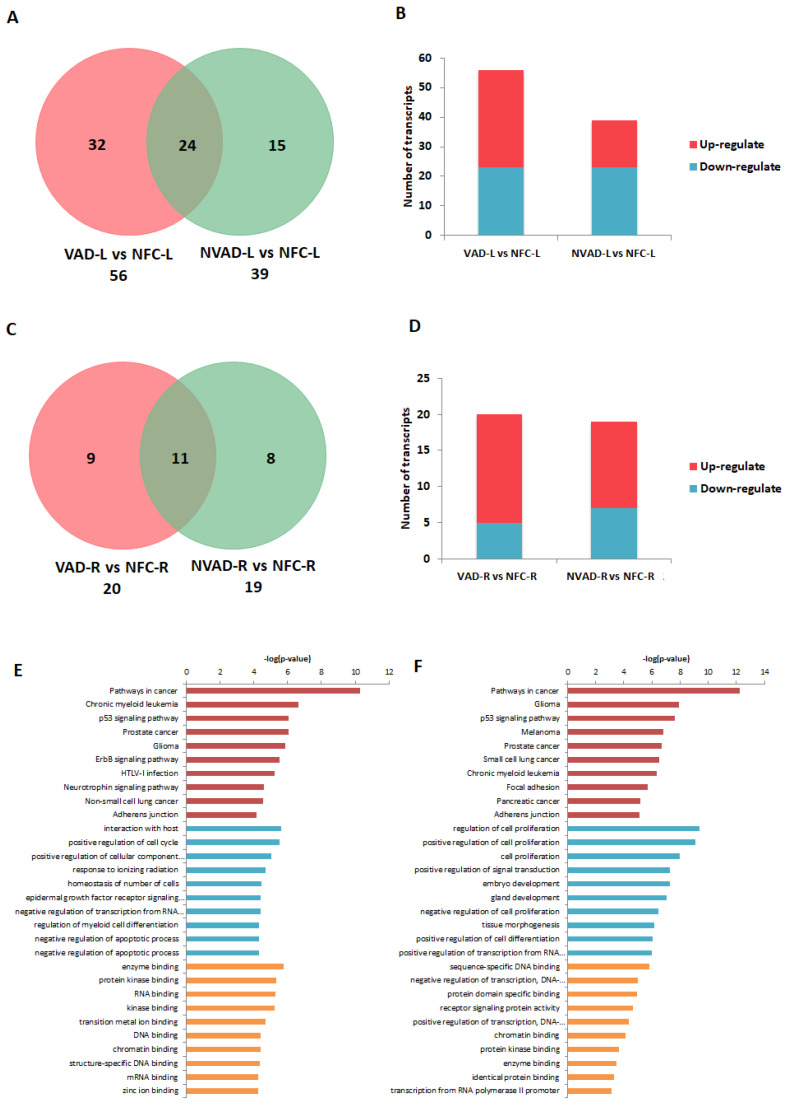
miRNA analysis of left (L) and right (R) ventricles from dilated cardiomyopathy (DCM) hearts with (VAD) and without left ventricle assist device (NVAD), using the non-failing controls (NFC) as reference. Number of significant differentially expressed miRNAs in the (**A**) L ventricle and (**C**) R ventricle of VAD and NVAD groups. (**B**) Number of significantly up- and down-regulated miRNAs in the (**C**) L ventricle and (**D**) R ventricle of VAD and NVAD groups. Gene ontology (GO) analysis for target genes of the differentially expressed miRNAs in the (**E**) L and (**F**) R ventricles following LVAD use. The top ten Kyoto Encyclopedia of Genes and Genomes (KEGG) pathways (red), GO terms for biological processes (blue) and GO terms for molecular functions are shown (yellow).

Similar to our previous analysis for the pathological mRNAs, the LV and RV pathological miRNAs (from NVAD vs. NFC) were assessed to examine how their expression levels changed upon LVAD implantation. The results from the VAD vs. NFC comparison revealed that of the 39 pathological miRNAs identified in the LV, 21 (54%) showed a decrease in FC with LVAD use compared to without LVAD use, and eight (21%) showed no change when compared to the NFC (Figure 6A–D; Table 4). On the other hand, of the 19 pathological miRNAs identified in the RV, 5 (26%) showed a decrease in FC with LVAD use compared to without LVAD use, and seven (37%) showed no significant change when compared to the NFC (Table 5). Comparisons between the VAD and NVAD groups showed that majority of pathological miRNAs were not normalized after LVAD implantation. One miRNA (hsa_miR_4458) in the LV and three miRNAs (hsa_miR_21*, hsa_miR_1972 and hsa_miR_4461) in the RV showed normalization. However, LVAD use also induced changes in the expression of other miRNAs (not from our “pathological” list) in both the LV (11 miRNAs) and RV (nine miRNAs). Enrichment analysis of the target genes of these miRNAs showed that the majority of them function in immunity and cellular process such as growth, proliferation and apoptosis (Figure 6E,F).

## 3. Discussion

**Figure 7:**
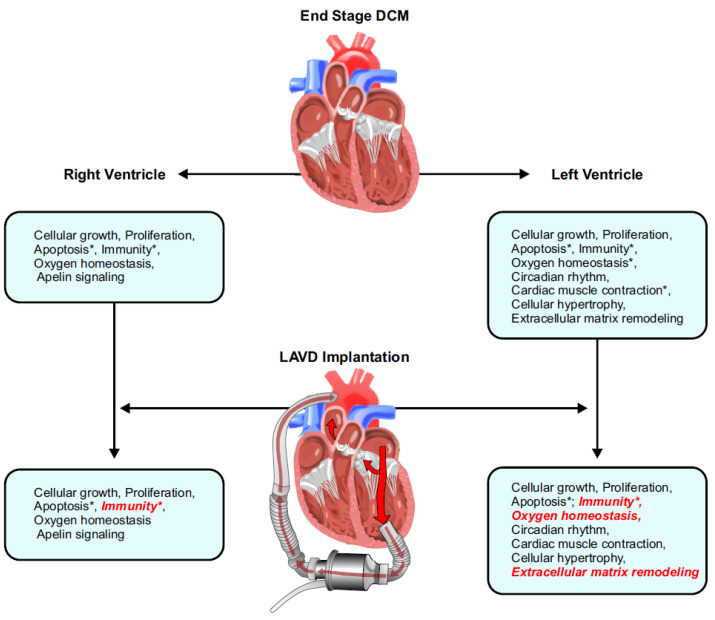
Summary of the findings from this study illustrating the seminal impact of LVAD therapy on reverse remodelling in the left ventricle and right ventricle. Processes that were normalized are italicized in red, and an asterisk (*) indicates pathways where the concerned genes also had corresponding changes at the miRNA level. DCM, dilated cardiomyopathy; LVAD, left ventricular assist device.

The main findings of our study are three-fold: (a) transcriptomic signatures of end-stage DCM hearts, (b) gene expression (mRNA) changes post-LVAD use, and (c) gene regulation (miRNA) changes post-LVAD use (Figure 7). The majority of differentially expressed genes in this study were down-regulated, suggesting a general loss-of-function model of pathogenesis and only the most significantly enriched transcripts in our dataset were considered. 

### 3.1. Pathological Genes Common in Both the LV and RV 

Cellular processes such as growth, proliferation, and apoptosis were significantly enriched among up-regulated genes in both the LV and RV. Genes that functioned in critical signalling pathways such as FoxO, JAK-STAT, AMPK, and Wnt were all up-regulated in end-stage DCM hearts. Genes involved in cell survival, cell cycle regulation, and energy metabolism are differentially expressed between DCM and healthy control hearts [15,16]. Cardiomyocyte apoptosis is common in patients with end-stage cardiomyopathy and has been reported by several studies [16,17]. Unlike most cell types, healthy postnatal cardiomyocytes are relatively non-proliferative and any cell loss could be detrimental to myocardial structure and function. Up-regulating genes and pathways involved in cellular growth and proliferation may, therefore, serve as a compensatory mechanism in the failing myocardium in the face of increased cell death.

On the other hand, genes involved in the immune response were most significantly enriched among the down-regulated genes. These functional gene classes included components of the complement system, neutrophil-mediated immunity, inflammatory response, and the humoral immune response. Activation of the immune system in heart failure is a widely known phenomenon [18,19]. The pro-inflammatory response in DCM is most likely a response to cardiomyocyte damage and often triggers cardiac fibrosis [18]. Furthermore, this inflammatory response activates the humoral immune system, which produces autoantibodies that can cause further tissue damage. Immune response-related genes displayed the most pronounced regulated genes in end-stage DCM [20]. Although down-regulation of genes involved in immune response was observed, our study confirms alterations in the immune system related genes and emphasizes the robust nature of the immune response in the DCM environment. Further characterization of how the immune system changes in the course of DCM may have implications in the treatment of DCM at various stages in its progression.

Genes involved in the HIF-1 pathway were also differentially expressed in both chambers of the DCM heart. HIF-1 controls oxygen delivery and utilization by regulating angiogenesis, vascular remodelling, and metabolic processes [21,22]. HIF-1 plays a protective role in the pathophysiology of ischemic heart disease and pressure-overload heart failure. HIF-1 activation has also been observed in association with the ischemic environment in DCM and over-expression of this pathway can lead to the development of cardiomyopathy [23,24]. We showed that HIF-1 was down-regulated in both LV and RV of end-stage DCM hearts suggesting that disturbance of oxygen homeostasis may play a role in the pathogenesis of end-stage DCM.

### 3.2. LV- and RV-Specific Pathological Genes

In the LV, the regulation of circadian rhythm is one of the most significantly enriched processes amongst up-regulated genes. Molecular circadian clocks exist in all cardiovascular cell types and various cardiovascular processes such as endothelial function, thrombus formation, blood pressure, and heart rate are under the regulation of the circadian clock [25]. Disruption of this rhythm results in cardiovascular diseases including heart failure, myocardial infarction, and arrhythmias. Disruption of the circadian rhythm leads to altered sarcomeric structure, cardiac fibrosis, and eccentric hypertrophy of myocardial walls, which eventually result in LV dilation and systolic dysfunction [26,27]. Our study is the first to show an association between dysregulated circadian rhythm and DCM at the transcript level in human hearts. Genes involved in cardiac muscle contraction were likewise enriched among up-regulated genes in the LV. Various processes such as myofibril assembly, sarcomere organization, or actin–myosin filament sliding were all up-regulated. LV transcriptomic and proteomic profiling in human end-stage DCM hearts found a large number of up-regulated pathways and processes belonging to the cardiomyocyte contractility family [15]. Up-regulation of genes involved in the cardiomyocyte compartment may serve to compensate for the impaired contractility observed in DCM. The cardiomyocyte hypertrophy pathway was also up-regulated in the LV of DCM hearts which is supported by our histological data. Our finding provides molecular evidence that pathological hypertrophy is a hallmark feature of DCM. 

ECM remodelling was enriched among the down-regulated genes in the LV. Remodelling of the ECM, particularly altered collagen homeostasis, has an important role in the pathogenesis of DCM [28,29,30]. In general, the rate of collagen turnover is controlled by the balance between matrix metalloproteinase (MMP) and tissue inhibitors of matrix metalloproteinase (TIMP) activity in the ECM [28,29,30]. However, TIMP expression in DCM hearts with some studies showing increased levels and others showing decreased levels compared to healthy controls suggesting a complex interplay between MMPs and TIMPs in the pathogenesis of DCM [28,29,30,31]. Our study showed decreased expression in *TIMP1*, *TIMP3,* and *TIMP4*, which potentially leads to decreased inhibition of MMPs and increased collagen turnover which was corroborated by our histological data.

In the RV, genes involved in the apelin signalling pathway were enriched among the up-regulated genes. The apelin/apelin receptor system functions in various cardiovascular processes such as vascular homeostasis, angiogenesis, cardiomyocyte contractility, cardiac hypertrophic response, and even in early cardiac development [32,33]. Our data for the first time, report significantly up-regulated apelin signalling pathway in the RV of end-stage DCM hearts. Whether this is an attempt of the failing heart to deal with the hypoxic environment and myocardial injury or up-regulation of the apelin pathway actually contributes to the deterioration of the end-stage DCM heart remains to be determined. The dynamic expression pattern of this pathway during disease development warrants further investigations. Our network analysis illustrated unique gene interactions at multiple levels in the LV and RV in patients treated medically and in those with LVAD and is consistent with an emerging view shaped by pre-clinical models that RV and LV have distinct embryological origins, workload and remodelling [34].

### 3.3. Gene Expression Post-LVAD Use

Upon LVAD implantation, genes involved in the immune response showed normalization (up-regulation) towards the healthy state in both the LV and RV. Proteomic analysis revealed increased abundance of innate immune response-related proteins, but a decreased abundance of complement system proteins [35]. However, it remains unknown if the up-regulation in immune response genes observed in this study is due to the unloading effect of LVAD on the ventricles or a triggered immune response by the insertion of a foreign object into the LV. Given the long duration of LVAD support in our study (median: 156 days, IQR: 131–268 days), perhaps the initial acute immune activation post-LVAD implantation plays less of a role in the observed up-regulation of immune response genes at the transcriptomic level. Moreover, our LV samples were taken at least 1 cm away from the insertion site of the LV cannula.

In the LV, genes involved in ECM remodelling were normalized after LVAD use. Both *TIMP1* and *TIMP3* (down-regulated in end-stage DCM without LVAD) were up-regulated post LVAD implantation. Besides gene normalization, LVAD use induced additional transcriptomic changes in the LV of the mechanically unloaded heart. Genes encoding for the alpha chain of collagen types 1, 3, and 14 (*COL1A1*, *COL3A1*, *COL14A1*) were all up-regulated in DCM hearts with LVAD compared to DCM hearts without LVAD. The effect of LVAD on the degree of collagen formation and fibrosis in the DCM heart is variable with some studies showing decreased collagen content post-LVAD, while others found increased collagen cross-linking with subsequent increased myocardial stiffness [36,37,38]. Although we observed an up-regulation of collagen expression, significant enrichment of TIMPs was also noted. TIMPs are suppressor of fibrosis and are known to decrease the deposition of excess collagen [28,39]. This complex interplay between the determinants of ECM remodelling can explain the overall reduction in fibrosis observed on staining. Our findings confirm the complex regulation of the ECM with up-regulation of TIMPs leading to increased inhibition of MMP activity and subsequently decreased ECM remodelling in the setting of reduced overall myocardial fibrosis.

Genes involved in the HIF-1 pathway for oxygen homeostasis were normalized (up-regulation) in the LV post-LVAD. Other genes related to angiogenesis were also up-regulated after LVAD implantation, which makes sense since the HIF-1 pathway was found to be a master regulator of angiogenesis [21,40]. Interestingly, the phosphatidylinositol 3-kinase (PI3K) pathway was up-regulated post-LVAD insertion and is known to play key roles in numerous cellular processes including angiogenesis and cytoskeletal remodelling [41,42]. Levels of heme oxygenase-1 (HO-1), a stress protein whose presence is induced by hypoxia, is decreased in DCM after LVAD use, suggesting improved myocardial hypoxia [43]. In our study, the normalization of genes involved in the HIF-1 pathway may contribute to the improved myocardial oxygen homeostasis following the unloading effect of LVAD. Pathways such as PI3K, MAPK, and TNFα were up-regulated after LVAD implantation. These pathways are important in numerous physiological processes such as the regulation of cellular metabolism, growth, proliferation, and apoptosis, which are critical mediators of the reverse remodelling from mechanical unloading of the failing heart via LVAD use [44,45].

### 3.4. Gene Regulation Post-LVAD Use

miRNA expression showed a trend towards normalization post-LVAD use, and four miRNAs (1 in the LV, 3 in the RV) were significantly normalized following LVAD implantation. In the LV, miR-4458 was significantly normalized following the unloading effect of LVAD. miR-4458 is a relatively new miRNA identified in several carcinomas [46,47]. At the cellular level, miR-4458 was shown to suppress cell proliferation and promote apoptosis in breast cancer and haemangioma [47,48]. miR-4458 functions as a negative modulator in cardiac hypertrophy and inhibition of this miRNA exacerbates cardiac hypertrophy. Our study is the first to demonstrate that miR-4458 is regulated in end-stage DCM with its expression normalizing after LVAD use. Our histological data support this finding, showing that cardiomyocyte CSA was normalized following LVAD implantation.

In the RV, 3 miRNAs (miR-21*, miR-1972, miR-4461) were significantly normalized post-LVAD use. While miR-1972 and miR-4461 have not been studied in the context of cardiovascular disease, miR-21* has been implicated in adverse myocardial remodelling [49,50]. Recent studies on miRNAs revealed the abundance of many miRNA* (miRNAstar), previously believed to undergo intracellular degradation [50]. MiR-21* induce hypertrophy of cardiomyocytes and inhibition of miR-21* alleviated cardiac hypertrophy in a model of angiotensin II-induced heart disease [51]. In contrast, another study revealed the intrinsic anti-hypertrophic function of miR-21* in cardiomyocytes [52]. Our study supports miR-21* role in the pathogenesis of heart failure and we found that LVAD use normalized the expression of this miRNA. LVAD use also induced changes in the expression of other miRNAs whose target genes function in the immune response and cellular physiological processes. While some studies found many miRNAs normalizing after LVAD use, others showed minimal changes [53]. Findings from our study suggest that changes in gene expression after LVAD implementation is partly attributable to miRNA regulation, and LVAD use has a more pronounced effect on miRNA normalization in the RV compared to the LV.

### 3.5. Strengths and Limitations

The limitations of our study include those of all descriptive studies using microarray or RNA sequencing. Microarray technology relies on a pre-determined set of probes so our study may not capture the full transcriptomic profiles of these hearts. However, proof of concept regarding gene expression and regulation generated in DCM hearts with or without LVAD should be further evaluated using next-generation sequencing [54]. Previous studies have examined molecular signatures in DCM hearts after LVAD use; however, the pre-LVAD samples were obtained from the apex region, while the post-LVAD samples were from the left ventricular free wall (LVFW) or septum. This regional variability between pre-and post-LVAD tissues may affect the evaluation of gene expression and regulation. The LVFW was consistently used in our study to control this regional variation. However, inter-individual variability may still play a role in our study since the pre-VAD and post-VAD samples were from different patients (as opposed to from the same people at the time of LVAD insertion and transplantation). The clinical background including mutations is an important modifier of myocardial gene expression. Future studies should expand analysis to these important variables to provide information on gene expression in the diseased human hearts. Another strength of our study is our ability to assess the transcriptomic changes in both the LV and RV of these hearts; in contrast, the majority of previous studies only looked at changes in the LV. We also studied both mRNA and miRNA expression and were, therefore, able to evaluate the effects of LVAD use on both gene expression and gene regulation, as well as assess whether changes in miRNA expression were accompanied by corresponding changes in mRNA expression. 

## 4. Materials and Methods

### 4.1. Study Patients and Protocol

The study protocol (Pro00011739) was reviewed and approved by the Health Research Ethics Board at the University of Alberta. The inclusion criteria were broad and included consecutive patients having heart transplantation at the Mazankowski Alberta Heart Institute and we were able to consent 99% of our patients. The only exclusion criterion was lack of written consent. All patients who participated in the study provided written informed consent in accordance with the Declaration of Helsinki. Clinical information for patients with DCM was collected via review of medical records from electronic databases maintained by Alberta Health Services based on the Human Explanted Heart Program (HELP) at the University of Alberta [4,54]. Available information on non-failing donor hearts was provided by the Human Organ Procurement and Exchange (HOPE) program in Northern Alberta.

### 4.2. Heart Tissue Procurement

LV and RV free-wall (LVFW and RVFW) tissue from two groups of explanted failing human hearts, DCM with LVAD (*n* = 8, male:female [M:F] = 7:1) or without LVAD (*n* = 8, M:F = 7:1) were collected via the HELP program. Non-failing control human hearts (*n* = 6, M:F = 4:2) were collected via the HOPE program. All hearts received cold cardioplegia, promptly explanted, preserved in cold saline, and kept on ice. Transmural myocardial tissue samples from the mid LVFW and RVFW were collected within 5 min of explantation and completed within 15 min, frozen in liquid nitrogen and stored at −80 °C for subsequent use [4].

### 4.3. Histology

#### 4.3.1. Picrosirius Red Staining

Formalin-fixed LVFW and RVFW were embedded in paraffin and sectioned with 5-μm thickness. Sections were stained using picrosirius red (PSR; Sigma, Oakville, ON, Canada) following standard procedure. Samples were visualized at 100× magnification using the Olympus IX-8 fluorescence microscope. Images were taken in at least 10 fields of view per sample to cover the whole slide. Myocardial collagen content was quantified using Metamorph Basic (Version 7.7.0.0). Values obtained across all fields of view were averaged for each sample. Image acquisition and myocardial collagen content quantification were conducted in a double-blinded fashion.

#### 4.3.2. Wheat Germ Agglutinin Staining

OCT-embedded frozen blocks of LVFW and RVFW were sectioned with 5 μm thickness. Sections were stained using Oregon Green 488–conjugated wheat germ agglutinin (Invitrogen, Burlington, ON, Canada) following standard procedure. Samples were visualized at 200× magnification using the Olympus IX-8 fluorescence microscope. Images were taken in at least 10 random fields of view per sample. Cardiomyocyte cross-sectional area was measured by tracing the area congruent to the wheat germ agglutinin-stained cardiomyocyte membrane using Metamorph Basic (Version 7.7.0.0). Values obtained across all fields of view were averaged for each sample. Image acquisition and cardiomyocyte cross-sectional area quantification were conducted in a double-blinded fashion.

### 4.4. RNA Processing and Microarray

RNA extraction was performed using an RNeasy Mini Kit (Qiagen, Toronto, ON, Canada) according to the manufacturer’s instructions. The purity of the total RNA was determined from the ratio of absorbance readings at 260 and 280 nm, with an A260/280 ratio between 1.8 and 2.0 indicating acceptable purity. RNA samples were stored at −80 °C for microarray analysis. BioAnalyzer was used to assess RNA sample quality. All RNA analysed had an RNA Integrity Number (RIN) of 7.5–10. Microarray analysis was performed using the PrimeView Human Gene Expression Array (Thermo Fisher, Ottawa, ON, Canada) and GeneChip miRNA 3.0 Array (Thermo Fisher), according to the manufacturer’s protocol. mRNAexpression analysis was performed using TaqMan reverse transcription polymerase chain reaction as before, using primers and probes obtained from Invitrogen [28]. mRNA levels for each gene were normalized to 18S levels for corresponding sample. 

### 4.5. Data Generation and Analysis

Transcriptomic data, both mRNA and miRNA, were normalized and analysed using Transcriptome Analysis Console (Version 4.0.1) (Thermo Fisher). One-Way Between-Subject ANOVA was used to calculate *p*-values, and a *p*-value of less than 0.05 was considered significant. Cut-off fold change (FC) for differentially expressed transcripts was set at 2 (≥2 for up regulation or ≤−2 for down-regulation). For analysis of transcript expression normalization after treatment with LVAD, only the *p*-value cut-off was applied in order to capture all effects of treatment on transcript expression. 

To identify significantly enriched functional pathways and gene ontology (GO) terms related to biological processes and molecular functions, differentially expressed genes were analysed using Enrichr (Available online: https://amp.pharm.mssm.edu/Enrichr/ (accessed on 7 January 2022)). The top ten most significantly enriched Kyoto Encyclopedia of Genes and Genomes (KEGG) pathways, GO terms related to biological process (BP), and GO terms related to molecular function (MF) were reported for each enrichment analysis. miRNet (Available online: https://www.mirnet.ca/miRNet/home.xhtml (accessed on 7 January 2022)) was used to predict miRNA target genes and their enrichment analysis. The top ten most significantly enriched KEGG pathways, GO terms related to BP, and GO terms related to MF were reported for the target genes, as previously performed. miRNA–mRNA interactions were analysed using Transcriptome Analysis Console (Version 4.0.1) (Thermo Fisher).

### 4.6. Statistical Analysis

Parametric continuous variables were expressed as means with their respective standard deviations (SDs). Non-parametric continuous variables were expressed as medians with their respective interquartile ranges (IQRs). Categorical variables were expressed as the total number in each category and the corresponding percentage of the study group they represented. Data distribution was assessed by the Kolmogorov–Smirnov test, D’Agostino–Pearson omnibus normality test, and Shapiro–Wilk normality test. All statistical analyses were performed using SPSS Statistics version 25 (IBM, New York, NY, USA). ANOVA followed by post hoc Tukey’s multiple comparison test, student’s t-test, two-tailed Mann–Whitney test, and two-sided Fisher’s exact test and were used for parametric continuous, non-parametric continuous, and categorical data, respectively.

## 5. Conclusions

Our study used microarray technology to determine the transcriptomic signatures of end-stage DCM hearts as well as the effects of LVAD use on these signatures. We identified major physiological processes contributing to the pathogenesis of end-stage DCM. Our results also suggest that the unloading effect of LVAD normalizes the expression of various genes and miRNAs towards healthy levels and that it also induces additional transcriptomic changes in the expression of both mRNAs and regulatory miRNAs. Right and left ventricular remodelling are distinct and we have identified multiple possible therapeutic targets for HF driven by LV and/or RV failure.

## Figures and Tables

**Table 1:** Clinical characteristics of DCM patients with and without LVAD implantation.

Criteria	No LVAD (*n* = 8)	LVAD (*n* = 8)	*p* Value
Age at transplant, years	50 (43–54)	57 (45–59)	0.7814
Female sex	1 (12.5)	1 (12.5)	>0.9999
SBP, mmHg	92 (89–97)	91 (80–103)	0.8131
HR, bpm	86 (69–106)	80 (77–96)	0.8935
BMI, kg/m2	24 (23–27)	25 (24–29)	0.3949
NYHA class			
III	6 (75)	4 (50)	0.5594
IV	1 (12.5)	3 (37.5)	0.5594
Medical history			
HF duration, mo	39 (6–108)	48 (30–120)	0.5493
Hypertension	1 (12.5)	1 (12.5)	>0.9999
Dyslipidemia	0 (0)	2 (25)	0.4667
Kidney disease	2 (25)	2 (25)	>0.9999
Liver disease	4 (50)	2 (25)	0.3147
Diabetes	0 (0)	1 (12.5)	>0.9999
COPD	1 (12.5)	1 (12.5)	>0.9999
Discharge medication			
ACEi/ARB/sacubitril/valsartan	7 (87.5)	6 (75)	0.3147
Beta-blocker	7 (87.5)	6 (75)	>0.9999
MRA	2 (25)	4 (50)	0.6084
Laboratory			
Cr, umol/L	108 (95–118)	83 (78–112)	0.9511
eGFR, mL/m in/1.73 m^2^	61 (56–69)	89 (65–112)	0.2811
Hb, g/L	118 (107–131)	98 (92–109)	0.1229
Echocardiography			
EF ≤ 50%	7 (87.5)	4 (50)	>0.9999
EF, %	18 (15–26)	13 (10–20)	0.6223
LVEDD, mm	64 (59–69)	57 (51–62)	0.3556
LVESD, mm	60 (54–66)	44 (38–59)	0.1523

SBP, systolic blood pressure; HR, heart rate; BMI, body mass index; NYHA, New York Heart Association; HF, heart failure; COPD, chronic obstructive pulmonary disease; ICD, implantable cardioverter defibrillator; CRT-D, cardiac resynchronisation therapy defibrillator; ACEi, angiotensin-converting enzyme inhibitor; ARB, angiotensin receptor blocker; MRA, mineralocorticoid receptor antagonist; Cr, creatinine; eGFR, estimated glomerular filtration rate; Hb, haemoglobin; EF, ejection fraction; LVEDD, left ventricular end diastolic diameter; LVESD, left ventricular end systolic diameter.

**Table 2:** Pathological miRNAs in the LV of DCM hearts and their corresponding target genes.

ID	Fold Change	*p*-Value	Target Transcripts from Corresponding mRNA Comparisons	
			Number of Transcripts	Target Genes
hsa-miR-451	16.22	4.30 × 10^−8^	43	*CCL18, FRZB, ACTC1, EIF1AY, C3AR1, RAMP1, AQP4, PLP2, F5, SLC35F1, KCNJ8, SLC25A27, LY96, STK38L, LDHA, TNFAIP8, EGLN3, ATP5F1, GPRASP1, UTY, SSPN, CTH, SORL1, GMNN, PTPRB*
hsa-miR-182	15.33	1.03 × 10^−6^	41	*IGF1R, DNER, BCAT1, CREB3L1, RARRES1, RNASE6, TPST2, HIF3A, EMB, MGST1, FCN3, MARK3, FIGF, MLF1, SLITRK4, BDNF, HS3ST1, SAMSN1, GABRB1, RSAD2, AASS, MYH6, ZMYND12, PKD1L2, TM2D3, DCN*
hsa-miR-495	2.87	1.14 × 10^−5^	30	*C3, CITED2, DNER, ALOX5AP, ACTC1, BCAT1, CRYM, AQP4, HSPA2, FAM46B, LARP6, CADM1, ATP5F1, GPR22, LRRTM4, RSAD2, AASS, DUSP27, AGTPBP1, PCDH20, HCLS1, TUBA1C, METTL7A*
hsa-miR-135b	−3.95	0.0003	30	*SERPINA3, CD53, GADD45A, FCER1G, RNASE6, IFIT2, ADH1A, FGF1, TIMP4, ANP32E, LILRB2, EFCAB2, RNASE2, CYP4Z1, ITIH5, IL1RL1, GABRB1, MAEL, RASAL2, HAS2, SLCO4A1, AGTPBP1, PTPRB*
hsa-miR-374b	2.35	0.0005	25	*F13A1, GADD45A, EGR1, CHST2, ASPN, CCL18, BIRC3, STK38L, ANKRD1, CTH, GOLGA8A, TMED5, ATP2A2, CNKSR2, PTPRB*
hsa-miR-218	4.42	0.0017	41	*PER3, FRZB, CRYM, PLCE1, IFI44L, DNAJB5, RABGAP1L, HAPLN1, SMTNL2, HEY2, ANKRD1, MLF1, CA2, ANK1, OGN, GABRB1, AASS, DUSP27, PHACTR1, GMNN, ST8SIA5, ASS1, UCHL1, RHOBTB1, DCN, EDIL3*
hsa-miR-208a	−2.58	0.0018	24	*SH3BGRL3, HMOX1, NPTX2, EMB, IFI44L, MGST1, TIMP4, LRRC1, MLF1, GPRASP1, C6, PDCD4, AGTPBP1, CPAMD8, HK2, BLM, TUBA1C*
hsa-miR-373	5.76	0.0026	27	*CD24, MAP4, NINJ2, PLCE1, MYBL1, C2orf40, GPR34, CD68, HS3ST1, CSAD, CNN1, ITIH5, GPRASP1, CHRFAM7A, SLMAP, SCUBE2, PRRT2, PKD1L2, UCHL1, IFI44*
hsa-miR-628-5p	2.59	0.0037	32	*CTSC, SMOC2, SLCO2A1, SLC1A2, BCL6, PLCE1, REPS2, OGDHL, PTN, DPY19L2, TYRP1, KCNIP2, C4A, MYOC, SCUBE2, GMNN, TSPAN13, BLM, PECAM1, TYROBP, ATP2A2*
hsa-miR-431	−2.33	0.0055	20	*CD53, SLC7A8, NPR3, LARP6, ITGAM, MYBPC1, STAT4, ITIH5, WISP2, GSG1L, CLEC10A, PHACTR1, ARPC3, CPAMD8, DDAH1*
hsa-miR-224	2.82	0.0058	30	*HLA-C, PACSIN3, TPST2, CYP2J2, HIF3A, GRB14, OGDHL, TMEM45A, HSD17B11, ARL4C, CHST9, CA2, GSG1L, C6, CTH, ARPC3, FHL1, HMOX2, ASS1, PTPRB*
hsa-miR-95	4.09	0.0069	36	*SERPINA3, C1QB, TUBB2A, PENK, TBXAS1, PLK2, CA14, TTC9, IL20RA, EDARADD, ST8SIA2, TFPI, CHST9, EGLN3, IL1RL1, GPRASP1, GSG1L, RSAD2, MGAT4C, ENO1, GMNN, PTPRB*
hsa-miR-940	−2.26	0.0078	17	*SH3BGRL3, SERPINA3, S100A9, NES, SERPINA1, HIF3A, GCHFR, CENPA, CYP4B1, CAPG, TNFRSF12A, IFI30*
hsa-miR-601	−2.29	0.0134	23	*PIM1, CD109, PLA2G2A, C1QC, SLC6A6, CYP2J2, GCHFR, CENPA, C1orf162, LILRB2, NR4A3, GRK5, TPM3, CA2, HMOX2*
hsa-miR-329	2.22	0.0148	25	*CITED2, HIF3A, AHNAK, KCNJ8, DPY19L2, CADM1, CD68, LRRTM4, PHACTR1, PRDX6, CEBPD, CAMK1D, NNMT, SLMAP, PKD1L2, AQP3, OBSCN*
hsa-miR-187	−6.09	0.0186	33	*SH3BGRL3, CHGB, S100A4, IGF1R, ALOX5AP, CYP2J2, PLP2, FAM58A, PRIMA1, MGST1, DPY19L2, HFE2, LILRB2, STK38L, STAT4, WISP2, GSG1L, DUSP27, PHACTR1, MXRA5, S100A8, CPAMD8, UCHL1, OBSCN*
hsa-miR-10b	2.55	0.0238	36	*APOD, FCER1G, RAMP1, DIO2, TMEM97, CA14, MYBL1, FAM46B, SMTNL2, GRK5, CD68, TNFRSF11B, LCN6, CNN1, ANK1, ATP5F1, SGPP2, C10orf71, PRDX6, SLMAP, SCUBE2, AGTPBP1, PKD1L2, OBSCN*
hsa-miR-223	2.99	0.0311	51	*GADD45A, PER3, AKR1C1, RRAS2, UBR1, PLCE1, RABGAP1L, MYO5A, TDRD9, GPR34, C1orf162, VIT, SLC14A1, EFCAB2, TFPI, ANK1, GPR22, OGN, MAEL, FKBP5, DUSP27, HVCN1, FHL1, CNKSR2*
hsa-miR-4269	2.38	2.70E-06	-	-
hsa-miR-299-5p	3.58	9.71 × 10^−5^	-	-
hsa-miR-4270	−2.12	0.0001	-	-
hsa-miR-4539	−2.06	0.0003	-	-
hsa-miR-1825	−2.04	0.0007	-	-
hsa-miR-3187-3p	−2.4	0.001	-	-
ENSG00000202498	−2.28	0.0013	-	-
ENSG00000202498_x	−2.31	0.0016	-	-
hsa-miR-3910	−2.03	0.0045	-	-
hsa-miR-4687-3p	−2.22	0.0057	-	-
hsa-miR-4741	−2.08	0.0071	-	-
hsa-miR-548x	−2	0.0077	-	-
hsa-miR-4689	−2.28	0.0107	-	-
hsa-miR-3128	−2.3	0.0109	-	-
hsa-miR-4793-3p	−2.69	0.011	-	-
hsa-miR-3201	−2.11	0.0114	-	-
hsa-miR-103b	−2.58	0.015	-	-
hsa-miR-1226	4.09	0.0158	-	-
hsa-miR-4458	2.21	0.0198	-	-
hsa-miR-4521	−3.93	0.0275	-	-
hsa-miR-1226	−2.12	0.0304	-	-

**Table 3:** Pathological miRNAs in the RV of DCM hearts and their corresponding target genes.

ID	Fold Change	*p*-Value	Target Transcripts from Corresponding mRNA Comparisons	
			Number of Transcripts	Target Genes
hsa-miR-182	19.89	6.64 × 10^−6^	48	*ANXA1, IL1R1, DNER, BCAT1, CREB3L1, RARRES1, RNASE6, PC, EMB, MGST1, APOB, FCN3, ACE2, MARK3, OLFM1, FIGF, CLEC4G, KCNN3, KCNMB2, PTGER3, SAMSN1, GABRB1, RSAD2, MYH6, ATP1B3, ZMYND12, MLF1, PKD1L2, CHI3L2, TM2D3, DCN*
hsa-miR-124	−2.16	0.0002	38	*CDKN1A, IQGAP1, IL1R1, CD59, RDH10, PENK, BCAT1, NAP1L3, GCHFR, AGTR1, KLF15, OLFM1, IRAK3, PAPSS2, CADM1, RNASE2, TRIM45, RGS4, RBM47, CYP4B1, NID1, SLCO4A1, ARPC1B, GOLGA8A, CPAMD8, TWIST2, C1S, TSC22D1*
hsa-miR-451	7.13	0.0004	38	*KLF6, HCK, CCL18, FRZB, ACTC1, C3AR1, RAMP1, AQP4, F5, CDKN2B, LY96, STK38L, B3GNT7, FYB, SCGB1D2, TNFAIP8, GPRASP1, MAN1A1, SSPN, CTH, SAT1, CTHRC1, ARPC1B, UTY*
hsa-miR-181a-2	2.34	0.0006	20	*VAMP8, RBP4, SOX4, IER3, ACLY, DIO2, ABCG2, C1orf162, CD163, SUSD4, ANKRD1, TPO, PHACTR1, NNMT, AQP3*
hsa-miR-373	6.81	0.0009	25	*CD24, KLF6, ENSA, NINJ2, PLCE1, UAP1, MYBL1, GPR34, CD68, MYPN, CNN1, ITIH5, GPRASP1, RASSF2, CHRFAM7A, IFI44, SCUBE2, PKD1L2*
hsa-miR-138	−2.39	0.0013	28	*LYZ, EIF4EBP1, FCER1G, CSTA, C3AR1, ADH1A, DOCK2, UAP1, APBB1IP, FIGF, VIT, TPM4, ITGAM, LCN6, SYNPO2L, TMEM74, STON1, PHACTR1, PKD1L2, C1S, DCN*
hsa-miR-431	−4.17	0.0016	24	*CD53, IGFBP6, HCK, SLC7A8, NPR3, LARP6, BMPR1B, HPR, ITGAM, MYBPC1, HP, ITIH5, TPO, CLEC10A, PHACTR1, CPAMD8, COL12A1*
hsa-miR-92a-1	−2.77	0.0025	29	*TIMP1, SH3BGRL3, SERPINA3, SEC14L1, AIF1, PLA2G2A, FCER1G, RAMP1, UAP1, METTL7B, LY96, GPX3, DPYSL4, ITIH5, PHACTR1, SAT1, CTHRC1, DUSP4, TKT, TYROBP*
hsa-miR-21	−2.87	0.0034	22	*SMOC2, IGFBP6, ALOX5AP, PENK, BCAT1, NQO2, BIRC3, CENPA, TDRD9, LARP6, TNFSF12, RGS4, C5AR1, ITIH5, MAP3K8, PKD1L2, ASRGL1*
hsa-miR-10b	2.64	0.0051	26	*ENSA, FCER1G, RAMP1, DIO2, CA14, HS6ST2, UAP1, MYBL1, SMTNL2, GRK5, CD68, SCN3A, LCN6, CNN1, C10orf71, SRGN, PRDX6, ARPC1B, SCUBE2, AGTPBP1, PKD1L2*
hsa-miR-95	2.86	0.0193	38	*SERPINA3, EMP1, C1QB, TUBB2A, PENK, TBXAS1, CA14, ACE2, EDARADD, ST8SIA2, CHST9, SHMT1, IL1RL1, GPRASP1, RSAD2, STON1, MGAT4C, SAT1, ENO1, CHI3L2, CHI3L1, GADD45B*
hsa-miR-217	3.1	0.0487	38	*CHST7, ENSA, HMOX1, MFAP5, KCNS3, FNDC1, CENPA, DOCK2, COL21A1, GFRA1, FCGR2A, FIGF, STRBP, CD163, HSPA4L, MXI1, MYPN, FGF10, STON1, DUSP27, MGAT4C, SAT1, CAMK1D, CPAMD8, PKD1L2*
hsa-miR-216a	3.02	0.0488	25	*KLF6, ENSA, FCER1G, NPR3, IFI44L, PITPNC1, STK17B, HFE2, VIT, CADM1, ITIH5, PTGER3, CTH, CYP4B1, ZMYND12, CCDC109B, HCLS1, CHI3L2*
HBII-52-32_x	4.76	0.0028	-	-
hsa-miR-1972	2.57	0.0046	-	-
hsa-miR-3065-3p	−3.04	0.0068	-	-
hsa-miR-4524	2.05	0.0101	-	-
hsa-miR-1247	−2.56	0.0187	-	-
hsa-miR-4461	2.3	0.0321	-	-

**Table 4:** Changes in pathological miRNA expression post-LVAD implantation in the LV.

ID	FC in NVAD vs. NFC	FC in VAD vs. NFC	*p*-Value in VAD vs. NFC
**Insignificant change compared to NFC**			
hsa-miR-4458_st	2.21	1.73	0.5994
hsa-miR-4793-3p_st	−2.69	−1.96	0.3478
hsa-miR-187_st	−6.09	−1.68	0.3441
hsa-miR-373_st	5.76	1.48	0.1852
hsa-miR-103b_st	−2.58	−1.81	0.096
hsa-miR-95_st	4.09	2.58	0.0677
hsa-miR-431_st	−2.33	−1.76	0.0659
hsa-miR-1226_st	4.09	2.2	0.064
**Decreased in FC**			
hsa-miR-182_st	15.33	9.68	3.58 × 10^−6^
hsa-miR-451_st	16.22	10.96	3.10 × 10^−8^
hsa-miR-3187-3p_st	−2.4	−1.37	0.0139
hsa-miR-548x_st	−2	−1.36	0.0076
hsa-miR-299-5p_st	3.58	2.95	0.0019
hsa-miR-4539_st	−2.06	−1.58	0.03
hsa-miR-628-5p_st	2.59	2.17	0.0047
hsa-miR-1226-star_st	−2.12	−1.76	0.0171
hsa-miR-4687-3p_st	−2.22	−1.91	0.0144
ENSG00000202498_x_st	−2.31	−2.08	0.0044
hsa-miR-223_st	2.99	2.8	0.0225
hsa-miR-10b_st	2.55	2.38	0.0145
hsa-miR-374b_st	2.35	2.21	0.0015
ENSG00000202498_st	−2.28	−2.16	0.0012
hsa-miR-4689_st	−2.28	−2.16	0.046
hsa-miR-1825_st	−2.04	−1.93	0.0033
hsa-miR-601_st	−2.29	−2.19	0.0158
hsa-miR-940_st	−2.26	−2.19	0.0282
hsa-miR-4741_st	−2.08	−2.02	0.005
hsa-miR-3910_st	−2.03	−1.99	0.0172
hsa-miR-4270_st	−2.12	−2.09	9.94 × 10^−5^

**Table 5:** Changes in pathological miRNA expression post-LVAD implantation in the RV.

ID	FC in NVAD vs. NFC	FC in VAD vs. NFC	*p*-Value in VAD vs. NFC
**Insignificant change compared to NFC**			
hsa-miR-1972_st	2.57	1.22	0.8742
hsa-miR-4461_st	2.3	1.14	0.7984
hsa-miR-21-star_st	−2.87	−1.27	0.647
hsa-miR-4524-star_st	2.05	1.32	0.3365
hsa-miR-373_st	6.81	1.66	0.0801
hsa-miR-431_st	−4.17	−3.62	0.0738
hsa-miR-1247_st	−2.56	−2.33	0.0583
**Decreased in FC**			
hsa-miR-182_st	19.89	11.3	1.75 × 10^−5^
hsa-miR-451_st	7.13	5.16	0.0002
HBII-52-32_x_st	4.76	3.48	0.0029
hsa-miR-181a-2-star_st	2.34	1.91	0.0098
hsa-miR-10b_st	2.64	2.53	0.0068

## Data Availability

Data available upon request.

## Supplementary Materials

The following supporting information can be downloaded at: https://www.mdpi.com/article/10.3390/ijms23042050/s1.
